# Supplementary material for: PRMT5-mediated regulatory arginine methylation of RIPK3
Source: Cell Death Discov. 2023 Jan 19;9:14. doi: 10.1038/s41420-023-01299-z (PMC9852244; doi:10.1038/s41420-023-01299-z)
Supplement: Supplementary file 2 — Supplementary Figures S1-S8 [file 41420_2023_1299_MOESM2_ESM.pdf]

Supplementary Data

(Supplementary Figures S1-S8)

PRMT5-mediated regulatory arginine methylation of RIPK3

Chanchal Chauhan[1], Ana Martinez Del Val[2], Rainer Niedenthal[1], Jesper Velgaard Olsen[2] Alexey Kotlyarov[1], Simon Bekker-Jensen[3], Matthias Gaestel[1*], Manoj B. Menon[4*]

[1]Institute of Cell Biochemistry, Hannover Medical School, Hannover, 30625, Germany.

[2]Mass Spectrometry for Quantitative Proteomics, Proteomics Program, The Novo Nordisk Foundation Center for Protein Research, University of Copenhagen, Copenhagen N, Denmark

[3]Center for Healthy Aging, Department of Cellular and Molecular Medicine, University of Copenhagen, 2200, Copenhagen, Denmark

[4]Kusuma School of Biological Sciences, Indian Institute of Technology Delhi, New Delhi, 110016, India.

*Correspondence to menon@bioschool.iitd.ac.in or gaestel.matthias@mh-hannover.de

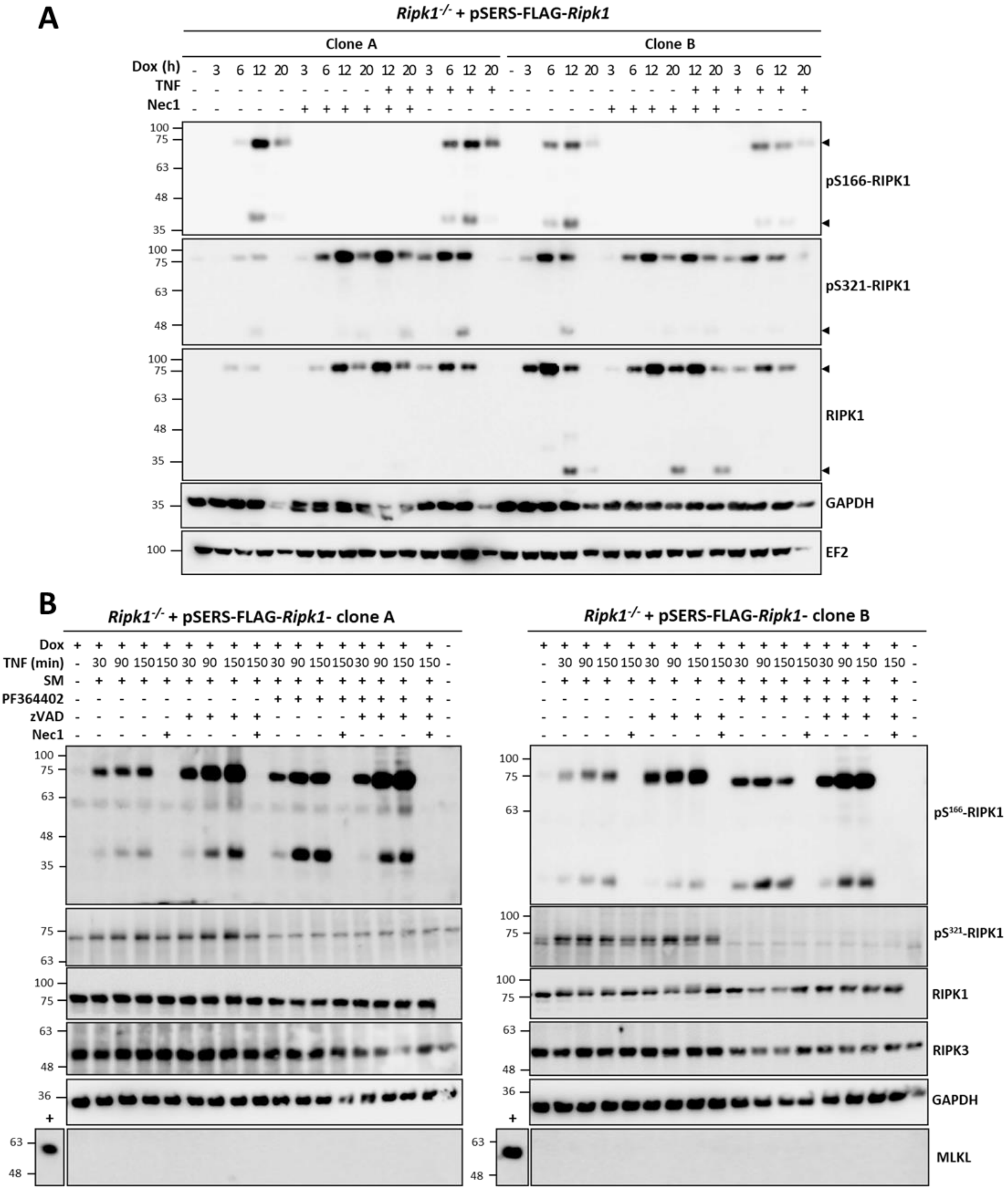

Supplementary Figure S1. Characterisation of RIPK1-activation kinetics in rescued Ripk1 KO cells. A. Two independent rescued Ripk1-KO cell lines (Clone A and B) were treated as indicated with doxycycline (Dox), followed by TNF stimulation in the presence and absence of the RIPK1 inhibitor necrostatin-1 (Nec1). Interestingly, long-term dox-treatment leads to spontaneous RIPK1 activation and subsequent cleavage of RIPK1 as indicated by pS166-RIPK1 immunoblots**. B.** The two clones were treated as indicated and the cell lysates were analysed with antibodies against pS166-RIPK1, pS321-RIPK1, RIPK1, RIPK3, GAPDH and MLKL. MLKL expression was not detected in these cells indicating lack of necroptosis. A positive control sample (MK2$^{-/-}$ mouse embryonic fibroblast lysates) was loaded to show that MLKL antibody was functional. The results shown are representative of two independent experiments.

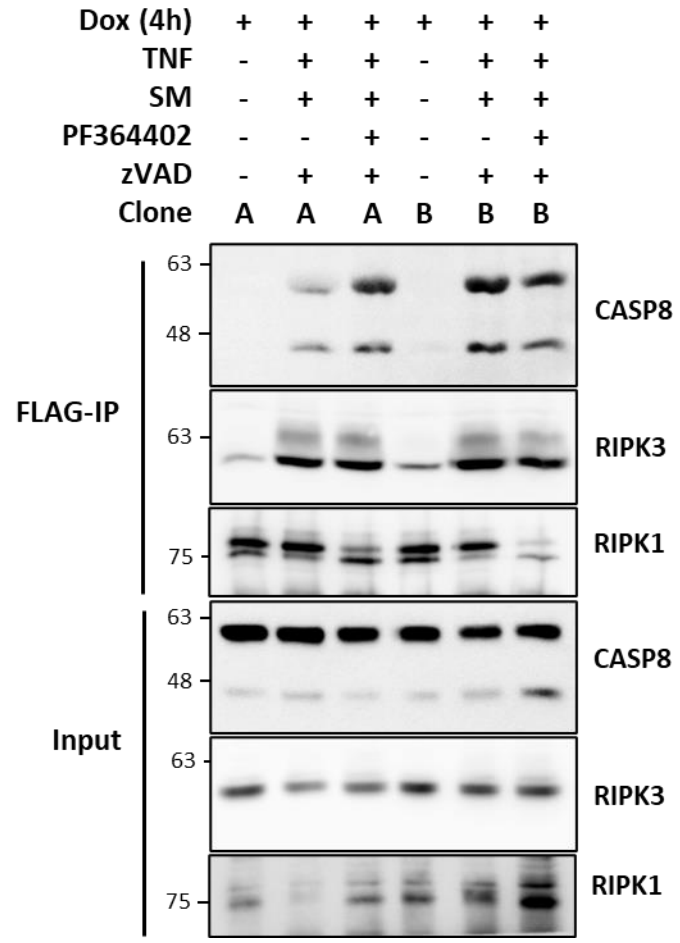

Supplementary Figure S2. RIPK3 is recruited to TNF-induced ripoptosome like complex. After addition of Doxycycline (Dox) for 4 hours, the rescued *Ripk1-KO* MEF cell lines (Clone A and B) were treated as indicated. Cell lysates were subjected to Flag-IP and blots were probed with antibodies against RIPK1, RIPK3, and CASP8 to monitor assembly of the ripoptosome.

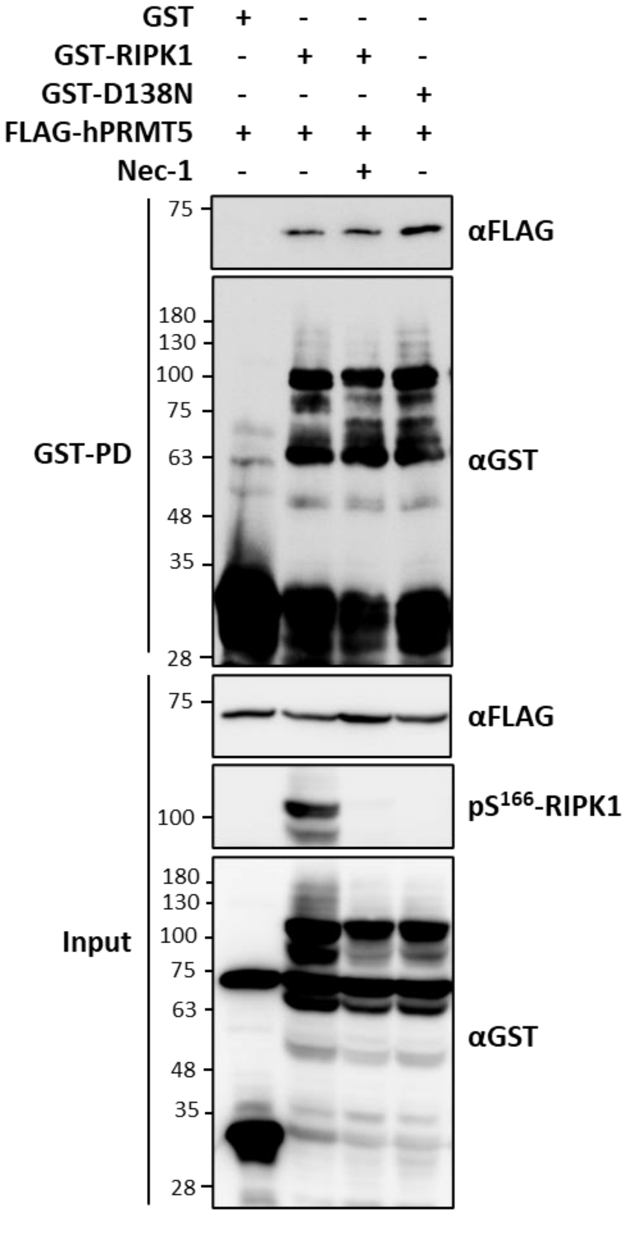

Supplementary Figure S3. RIPK1 activity is dispensable for association with PRMT5. GST-pulldown assay from transfected HEK293T cells shows specific interaction of PRMT5 with WT-RIPK1 in presence or absence of RIPK1 activity inhibitor (Necrostatin-1) as well as with catalytic-deficient RIPK1 mutant (RIPK1-D138N). The results shown are representative of three independent GST-pulldown assays.

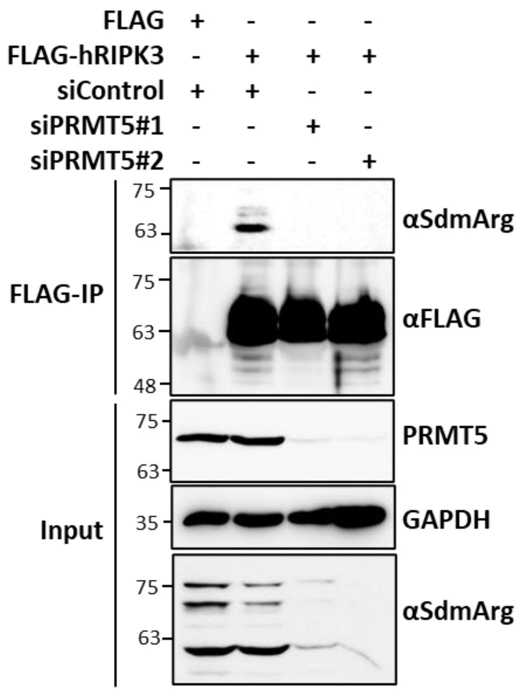

Supplementary Figure S4. PRMT5 knockdown abrogated hRIPK3 methylation. FLAG-tagged human RIPK3 was immunoprecipitated from HEK293T cell transfected with siRNAs targeted against PRMT5 or control siRNA. The samples were probed with anti-symmetric dimethyl arginine antibodies. Control blots show efficient knockdown of PRMT5 and effective downregulation of general methylation signals in the input cell lysates. The results shown are representative of two independent knockdown experiments

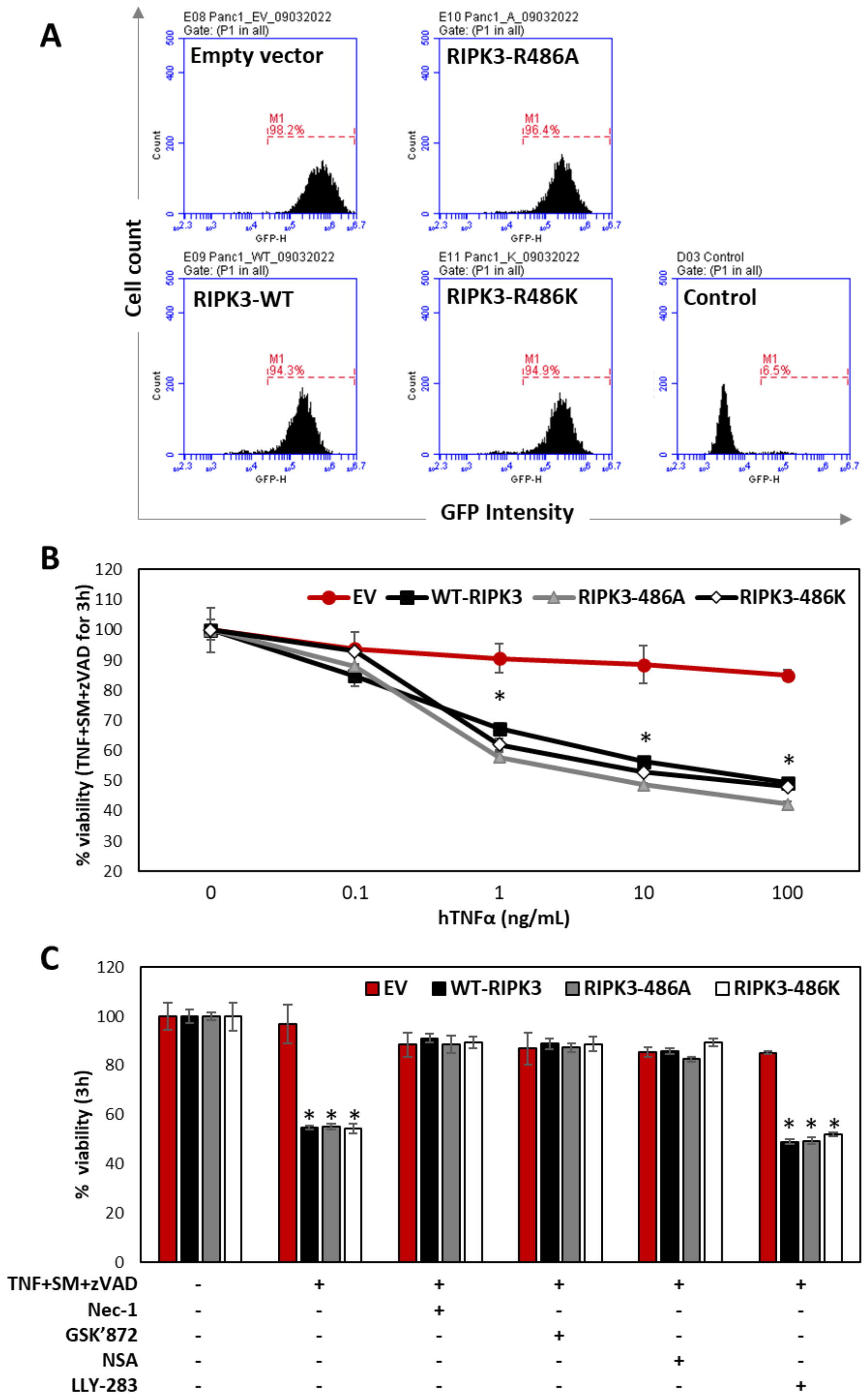

Supplementary Figure S5. Characterisation of RIPK3-rescued PANC1 cells. A. PANC1 cells transduced with empty vector (EV) and WT/mutant RIPK3 expression vectors were selected with puromycin, followed by uniform GFP marker expression quantified by flow-cytometry. **B.** Expression of RIPK3 sensitized PANC1 cells to necroptosis. When cells were treated with increasing doses of hTNFα for 3h, in the presence of smac-mimetics (SM) and zVAD, the rescued cells displayed significant loss of viability compared to empty vector transduced PANC1 cells. **C.** As in panel B, TNF/SM/zVAD treatment (10ng/mL TNF) induces necroptosis in RIPK3-transduced PANC1 cells. Treatment with necrostatin-1 (Nec-1, RIPK1 inhibitor), GSK'872 (RIPK3 inhibitor) or necrosulfonamide (NSA, MLKL inhibitor) inhibited cell death establishing the process and necroptosis, while PRMT5 inhibitor (LLY-283) did not show any effect. The death assay results shown are representative of three independent experiments. Statistical analysis by two-tailed unpaired *t*-test (n=3) and * denotes p-value<0.01. (Source data and actual *p*-values in Supplementary table 3)

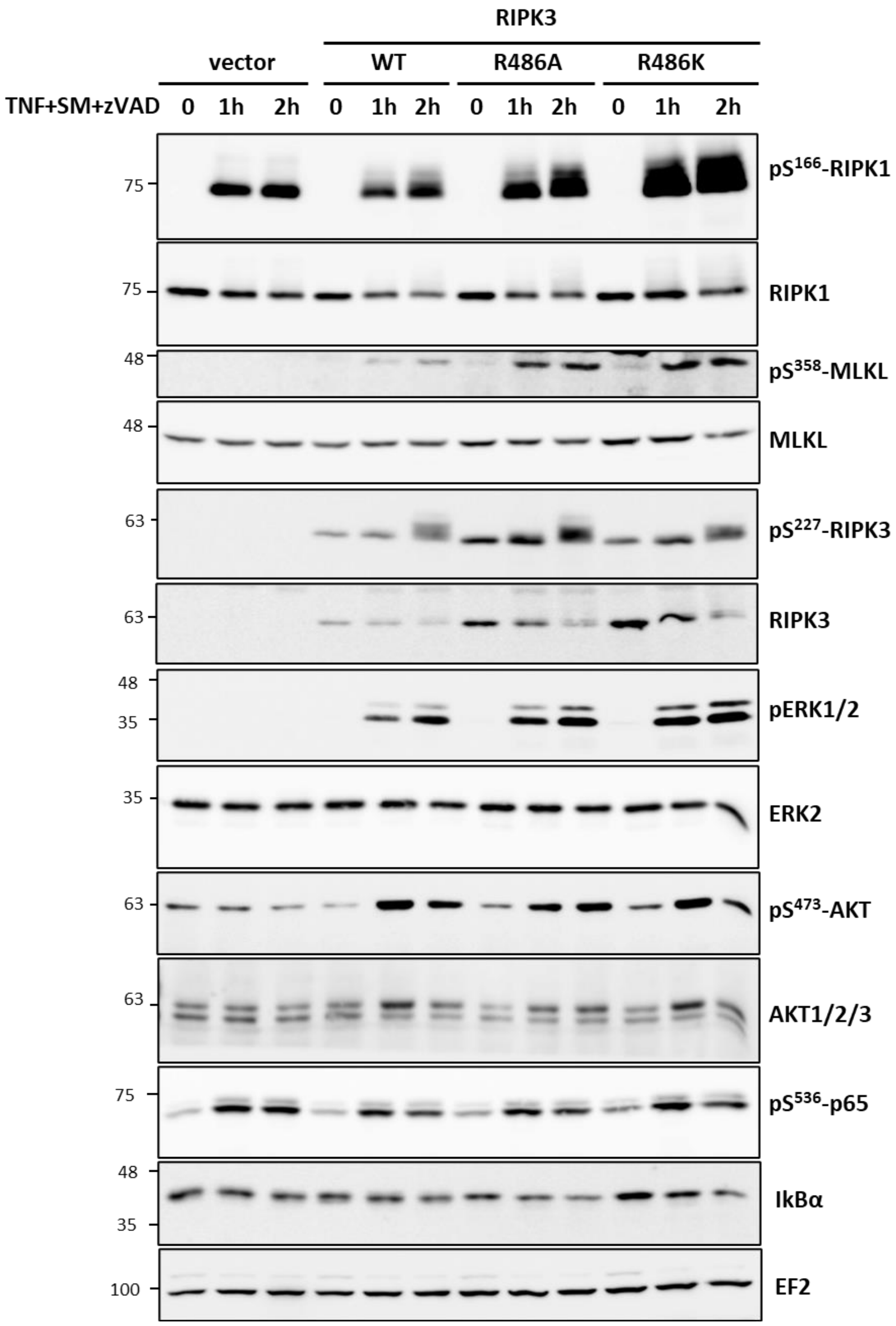

Supplementary Figure S6. Necroptotic signaling in RIPK3-rescued PANC1 cells.
PANC1 rescue model as in Figure 4, panel B comparing the necroptotic signaling in cells transduced with WT and methylation site mutants (R486K and R486A) of FLAG-tagged RIPK3. The figure depicts the signaling effects and the processes targeted by the inhibitors used. RIPK3-mediated suppression of RIPK1-S166 is lost upon methylation site mutation. In addition, the necroptotic signaling is also enhanced in the absence of R486 methylation. In contrast pS^{473}AKT remain unaffected. The results shown are representative of three independent experiments

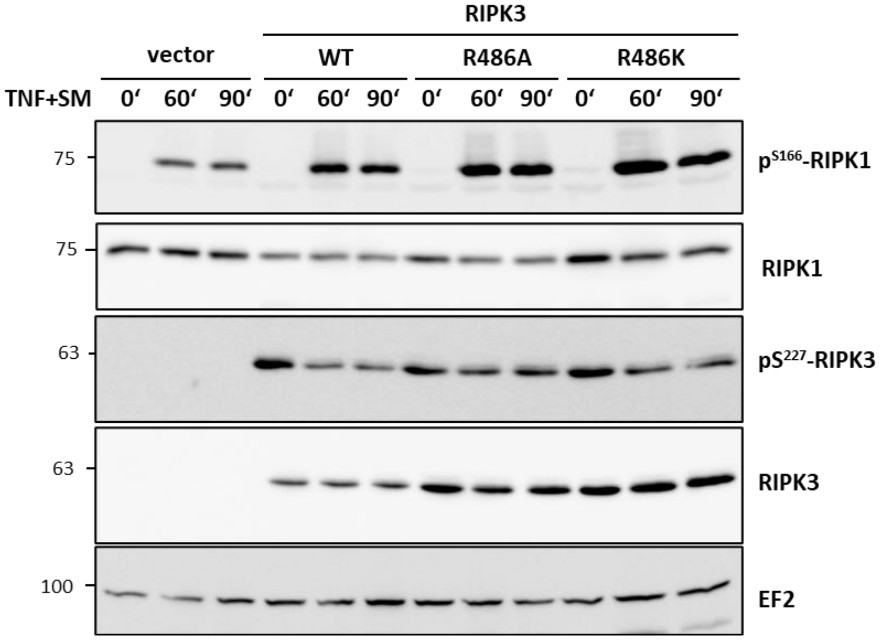

Supplementary Figure S7. Role of RIPK3 in RIPK1 autophosphorylation in response to pro-apoptotic stimulus. PANC1 rescue model as in Figure 4A comparing the apoptotic signaling in cells transduced with Wild-type and methylation site mutants (R386K and R386A) of FLAG-tagged human RIPK3. The cell lysates were probed with indicated antibodies after Immunoblotting to detect RIPK1 and RIPK3 activation and levels. EF2 is shown as sample-loading control. The results shown are representative of three independent experiments

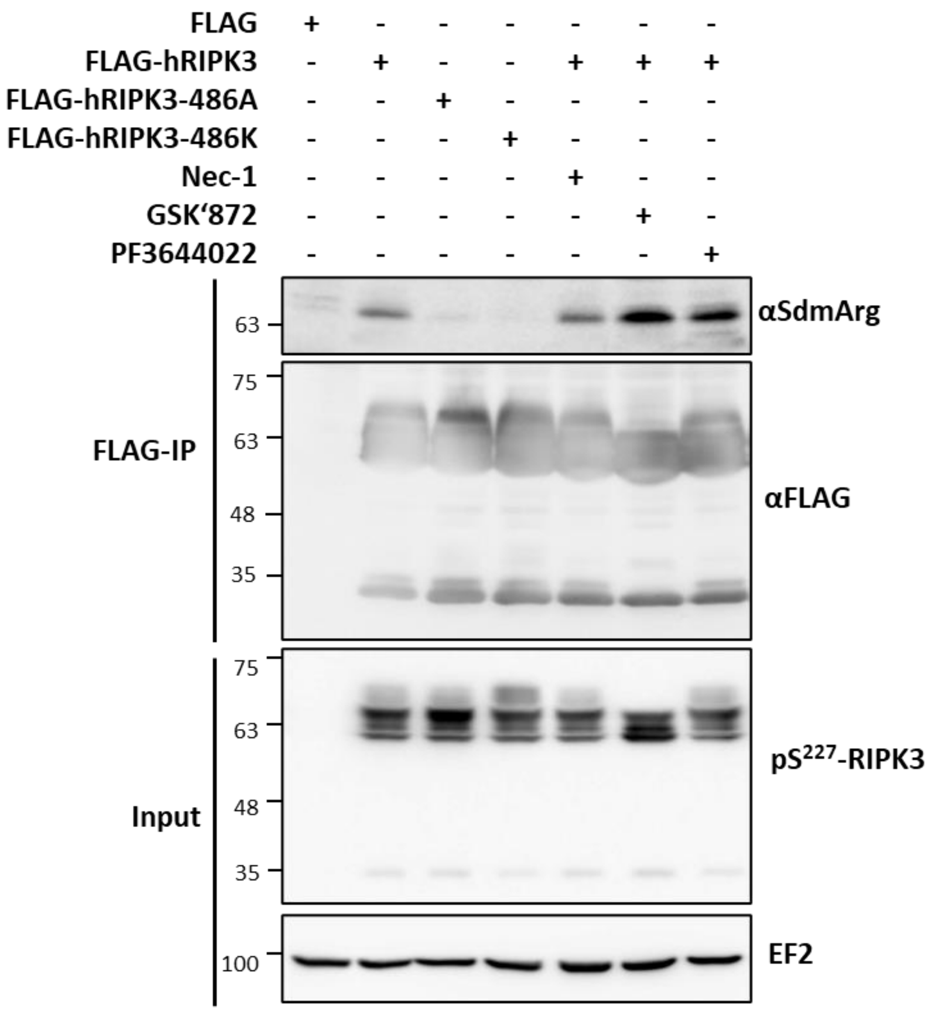

Supplementary Figure S8. Kinase activity of RIP kinases are not required for methylation of RIPK3. Effect of RIPK1 and RIPK3 inhibitors Necrostatin-1 (Nec1) and GSK'872 on RIPK3 methylation monitored by GST-enrichment and immunoblotting in HEK293T Cells. The results shown are representative of two independent experiments
