# Supplementary material for: PRMT5-mediated regulatory arginine methylation of RIPK3
Source: Cell Death Discov. 2023 Jan 19;9:14. doi: 10.1038/s41420-023-01299-z (PMC9852244; doi:10.1038/s41420-023-01299-z)

Fig. 1A

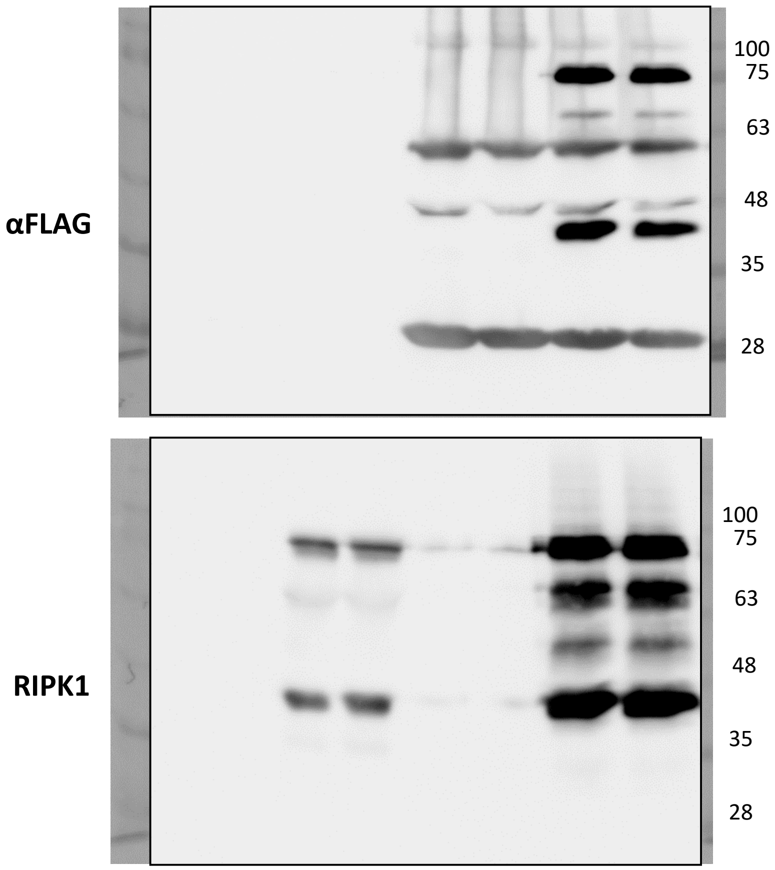
αFLAG
RIPK1
100
75
63
48
35
28
100
75
63
48
35
28

Fig. 1B

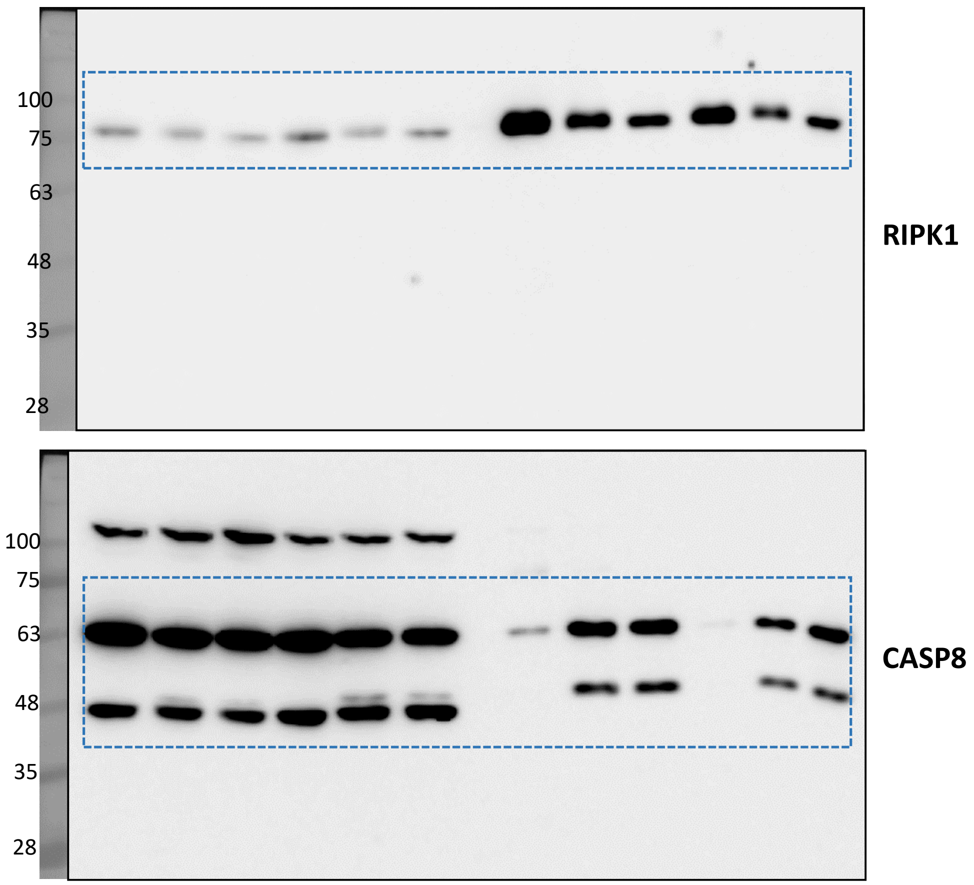
RIPK1
CASP8
100
75
63
48
35
28
100
75
63
48
35
28

Fig. 1D

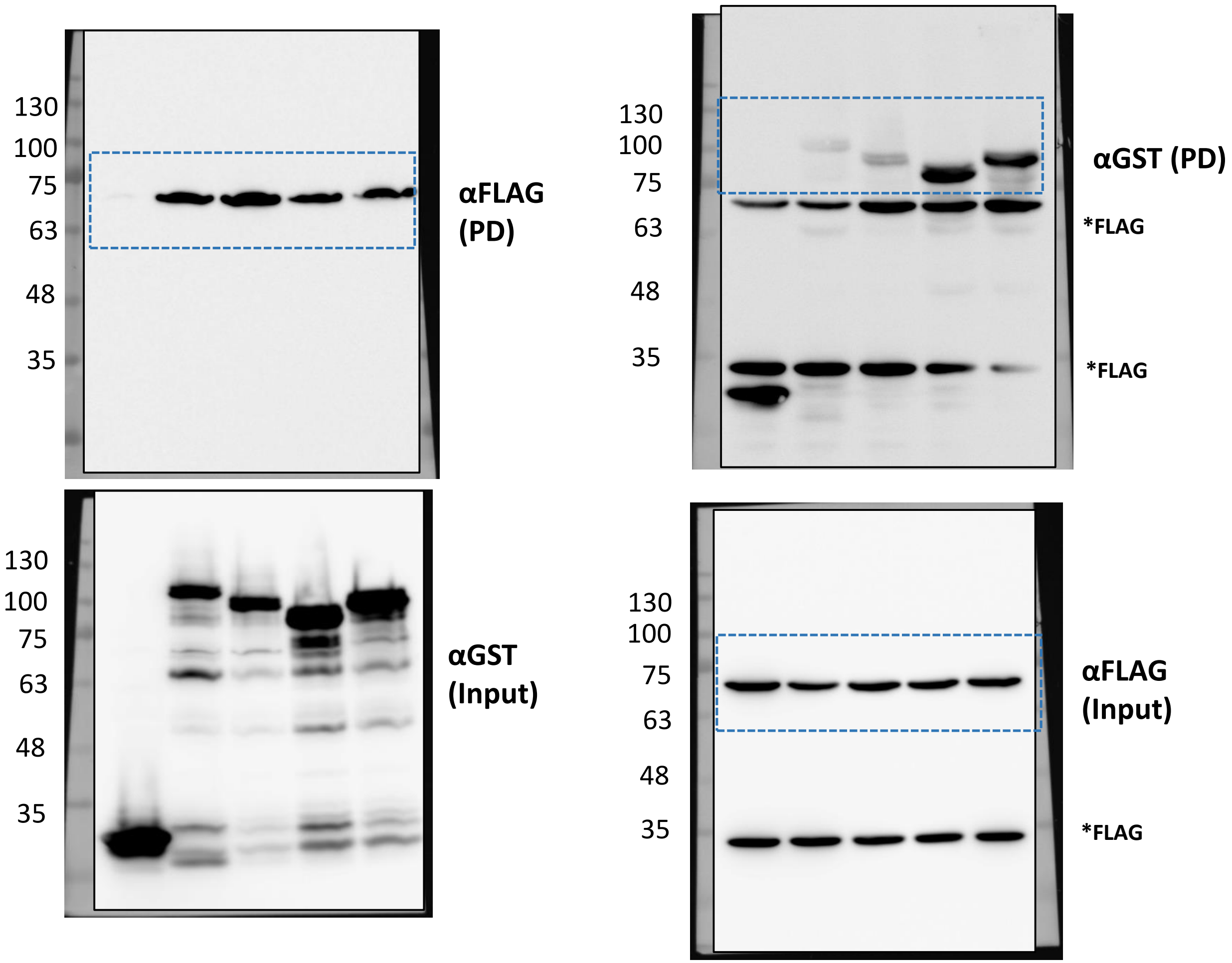

Fig. 2A

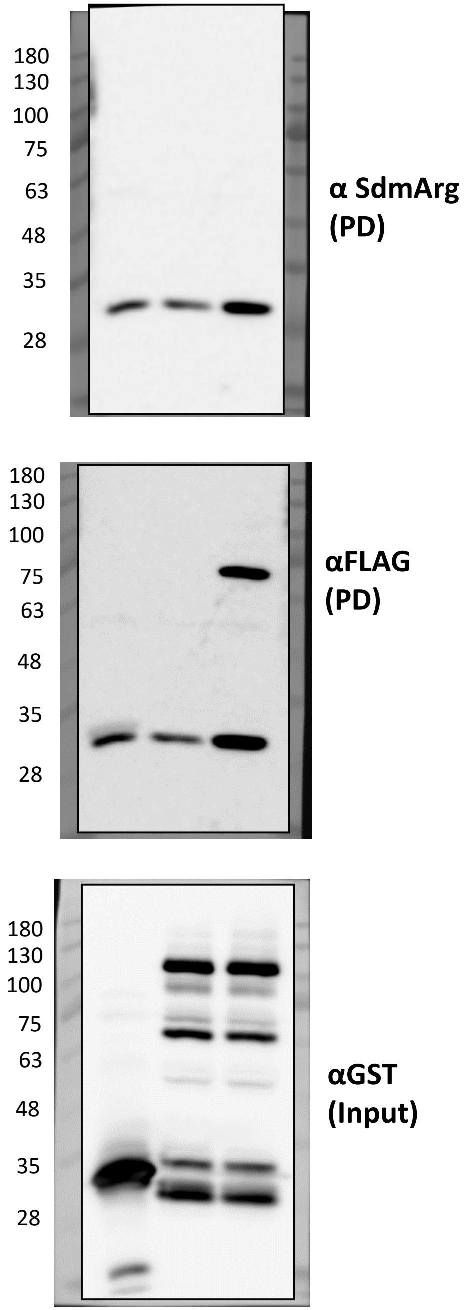

Fig. 2B

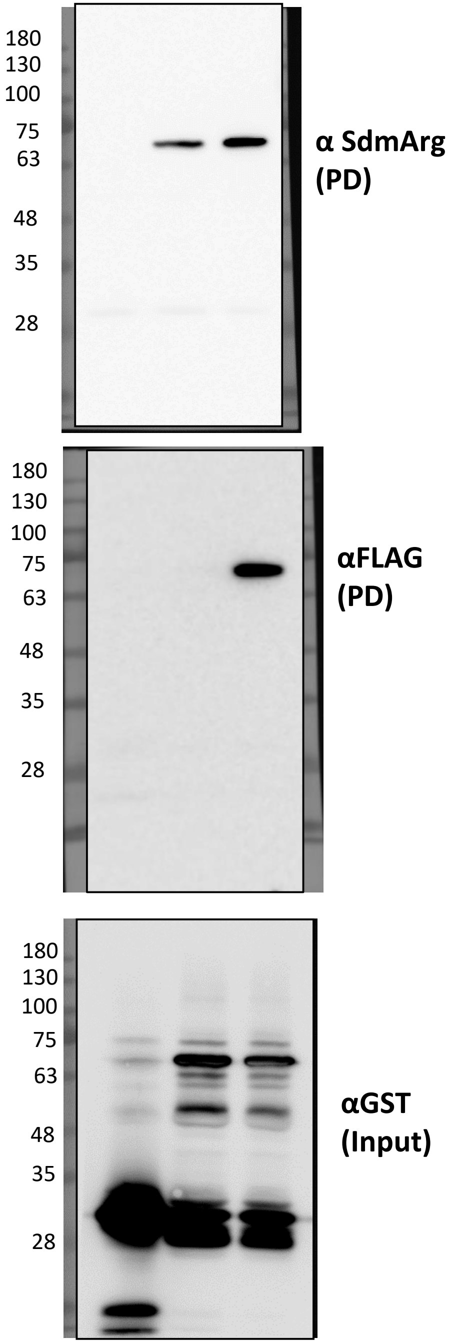

Fig. 2C

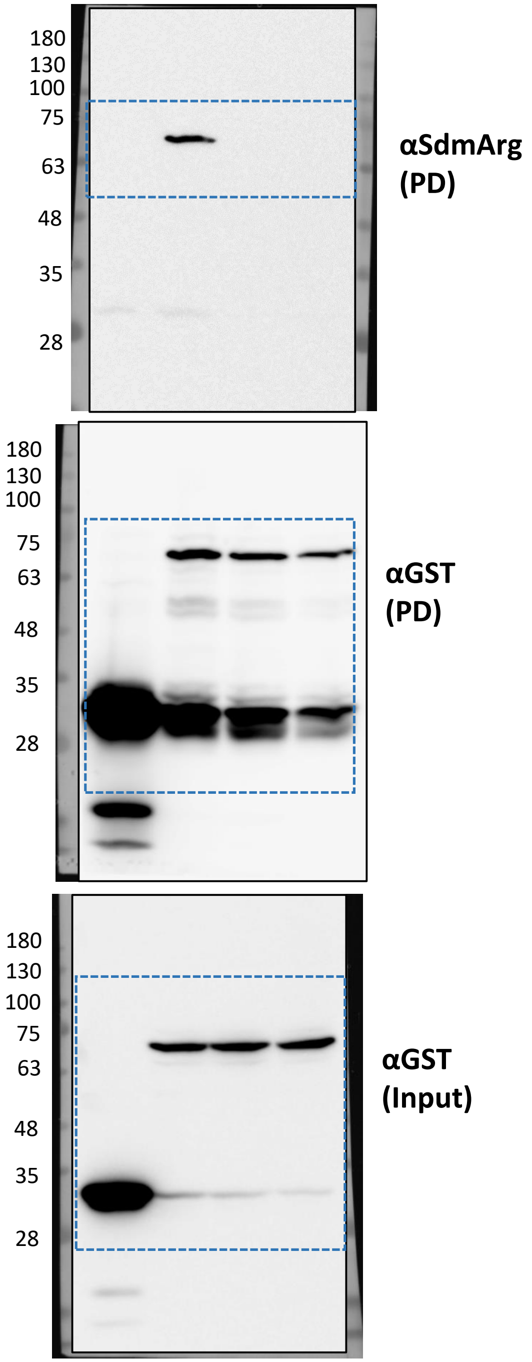
180
130
100
75
63
48
35
28
αSdmArg
(PD)
180
130
100
75
63
48
35
28
αGST
(PD)
180
130
100
75
63
48
35
28
αGST
(Input)

Fig. 2D

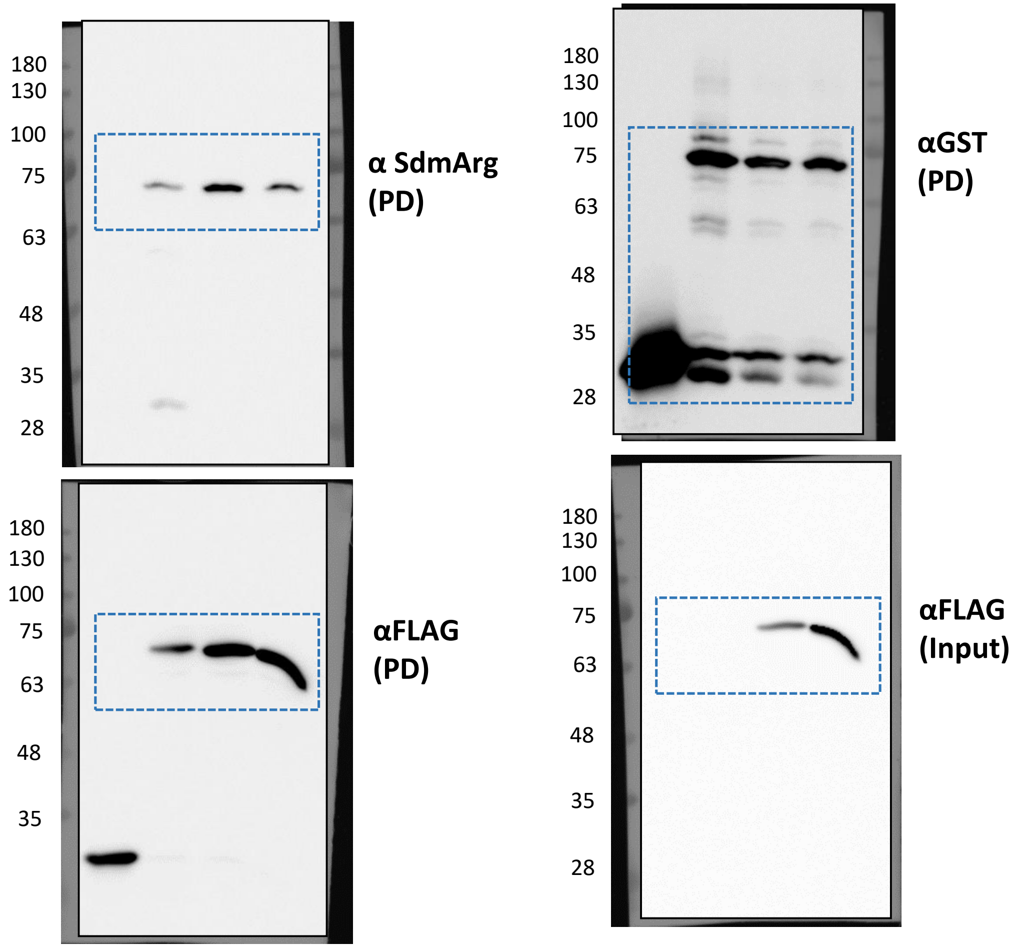
180
130
100
75
63
48
35
28
α SdmArg
(PD)
180
130
100
75
63
48
35
αFLAG
(PD)
180
130
100
75
63
48
35
28
αGST
(PD)
180
130
100
75
63
48
35
28
αFLAG
(Input)

Fig. 2E

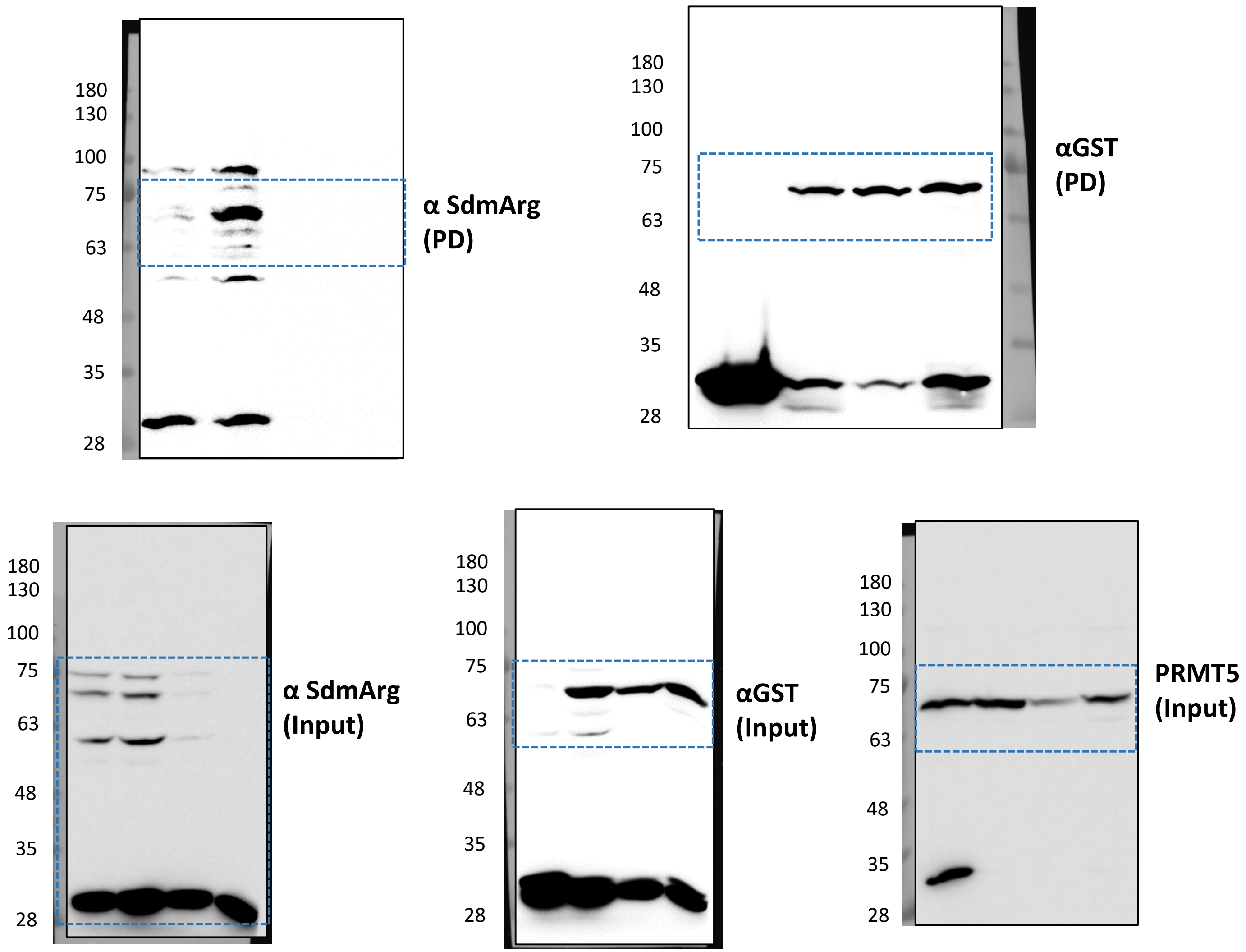
180
130
100
75
63
48
35
28
α SdmArg
(PD)
αGST
(PD)
α SdmArg
(Input)
αGST
(Input)
PRMT5
(Input)

Fig. 3B

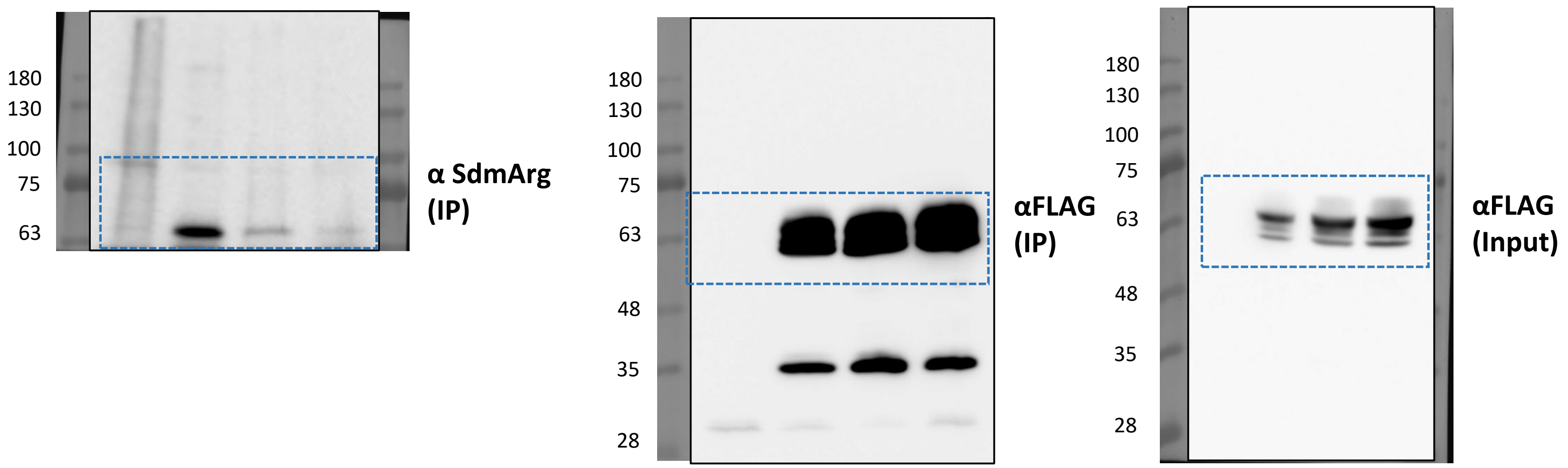
180
130
100
75
63
α SdmArg
(IP)
180
130
100
75
63
48
35
28
αFLAG
(IP)
180
130
100
75
63
48
35
28
αFLAG
(Input)

Fig3D

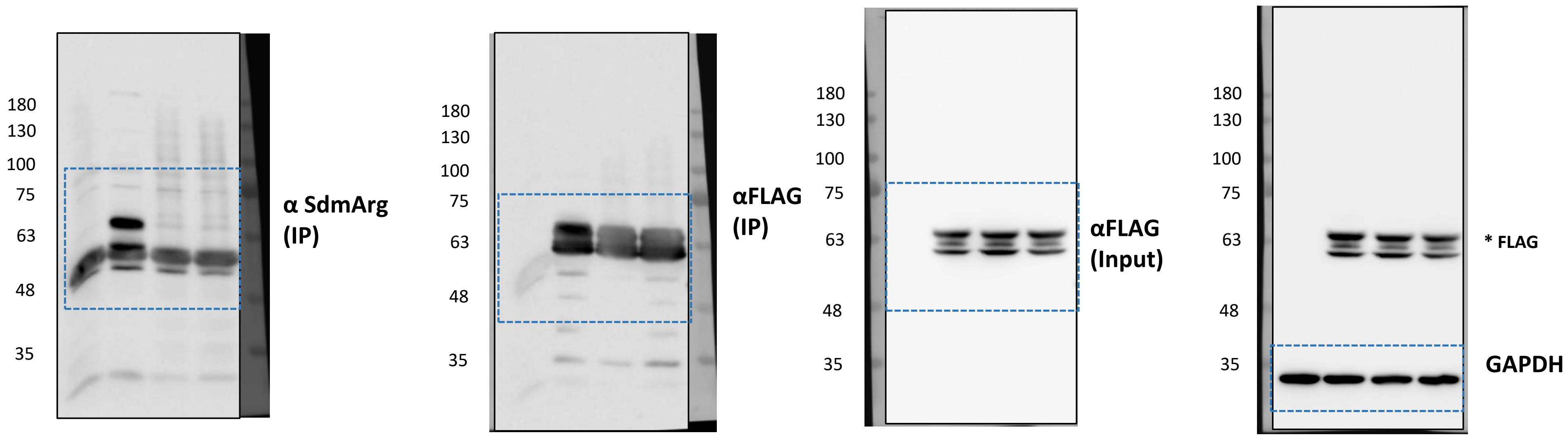
180
130
100
75
63
48
35
α SdmArg
(IP)
180
130
100
75
63
48
35
αFLAG
(IP)
180
130
100
75
63
48
35
αFLAG
(Input)
180
130
100
75
63
48
35
* FLAG
GAPDH

Fig. 4A

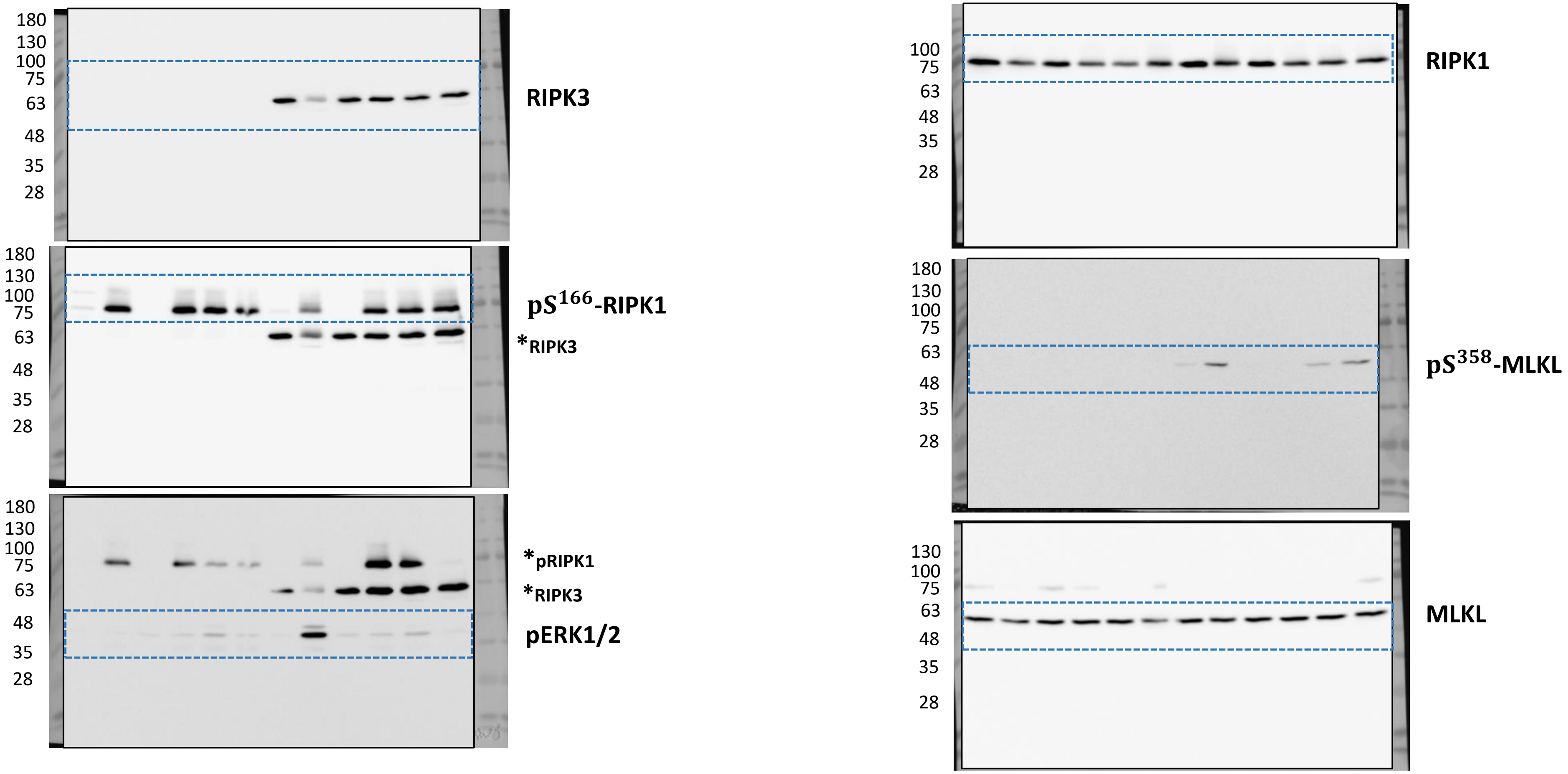
180
130
100
75
63
48
35
28
RIPK3
pS^{166}-RIPK1
*RIPK3
*pRIPK1
*RIPK3
pERK1/2
RIPK1
pS^{358}-MLKL
MLKL

Fig. 4B

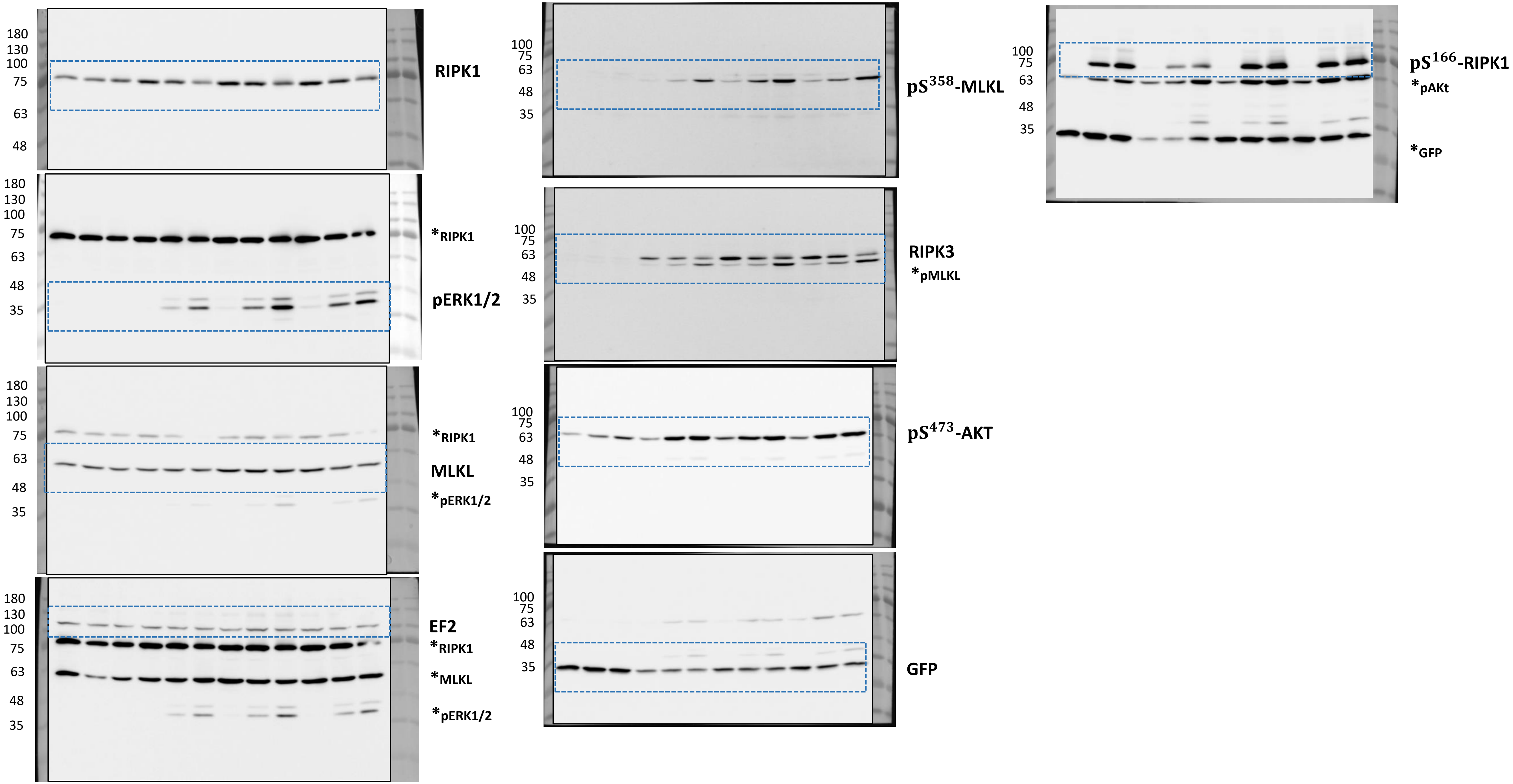

180
130
100
75
63
48
35
RIPK1
*RIPK1
pERK1/2
MLKL
*pERK1/2
EF2
*MLKL
pS358-MLKL
RIPK3
*pMLKL
pS473-AKT
GFP
pS166-RIPK1
*pAKt
*GFP

Supplementary Fig. S1A

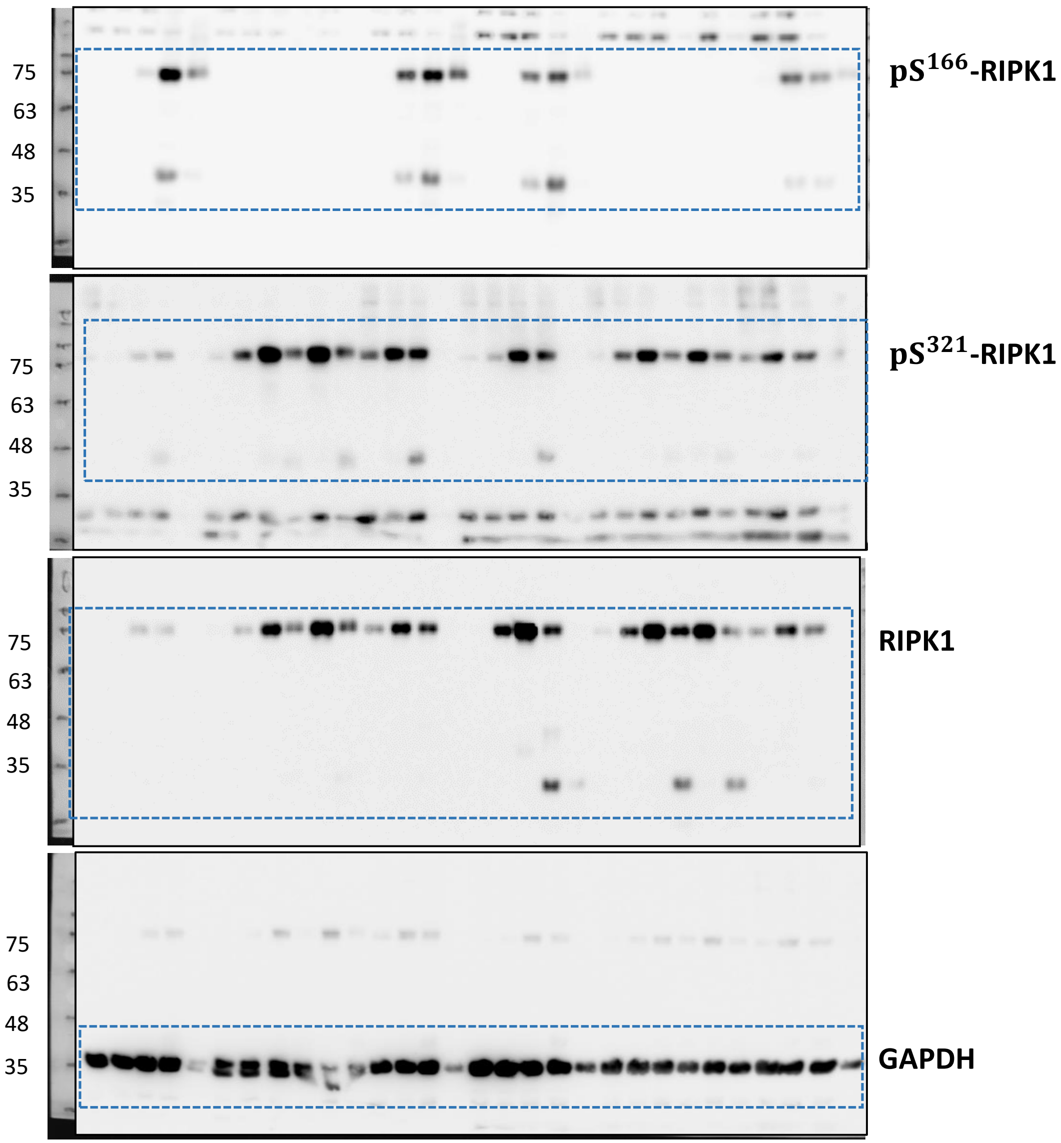

Supplementary Fig. S1B

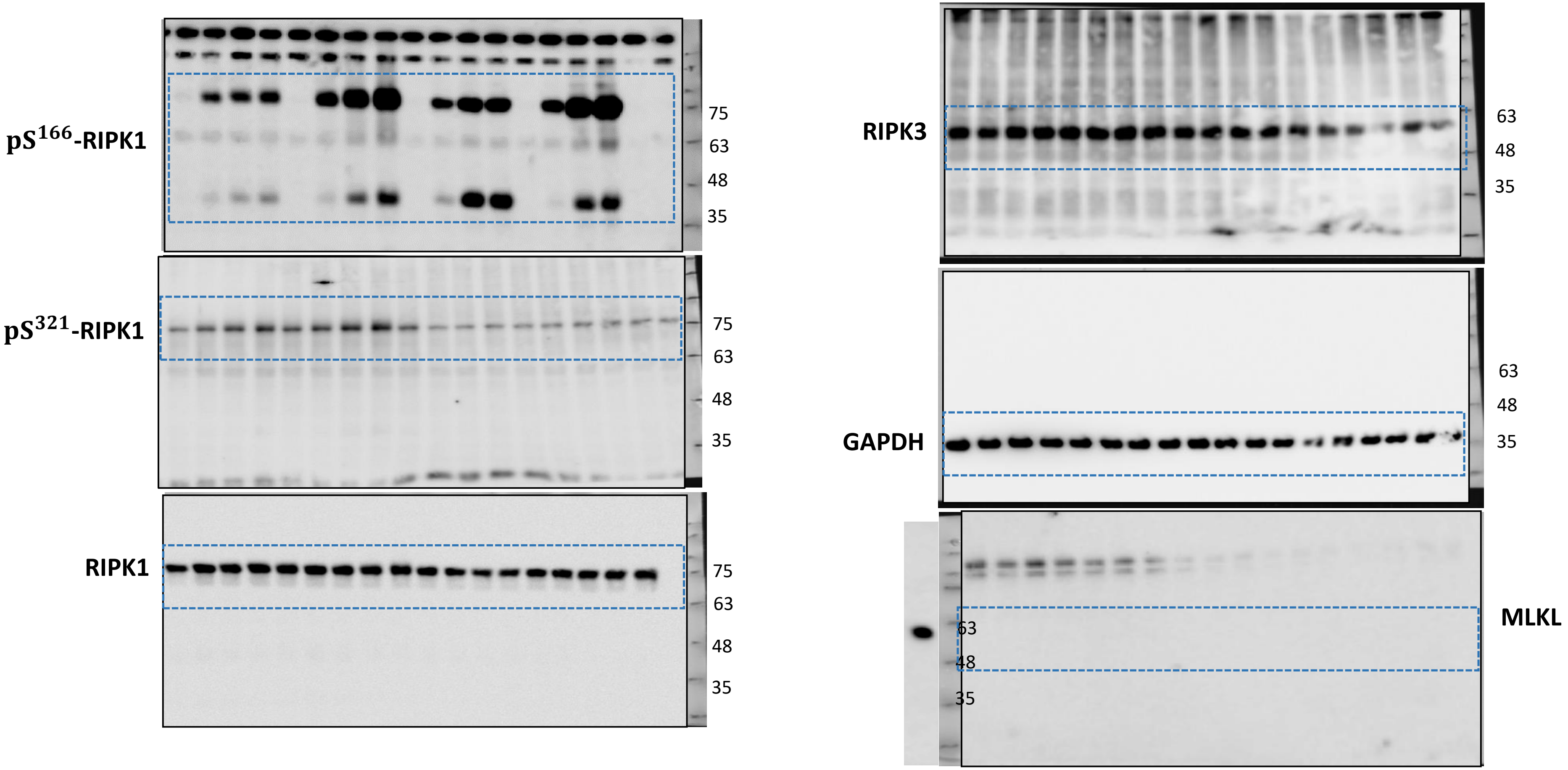

Supplementary Fig. S1C

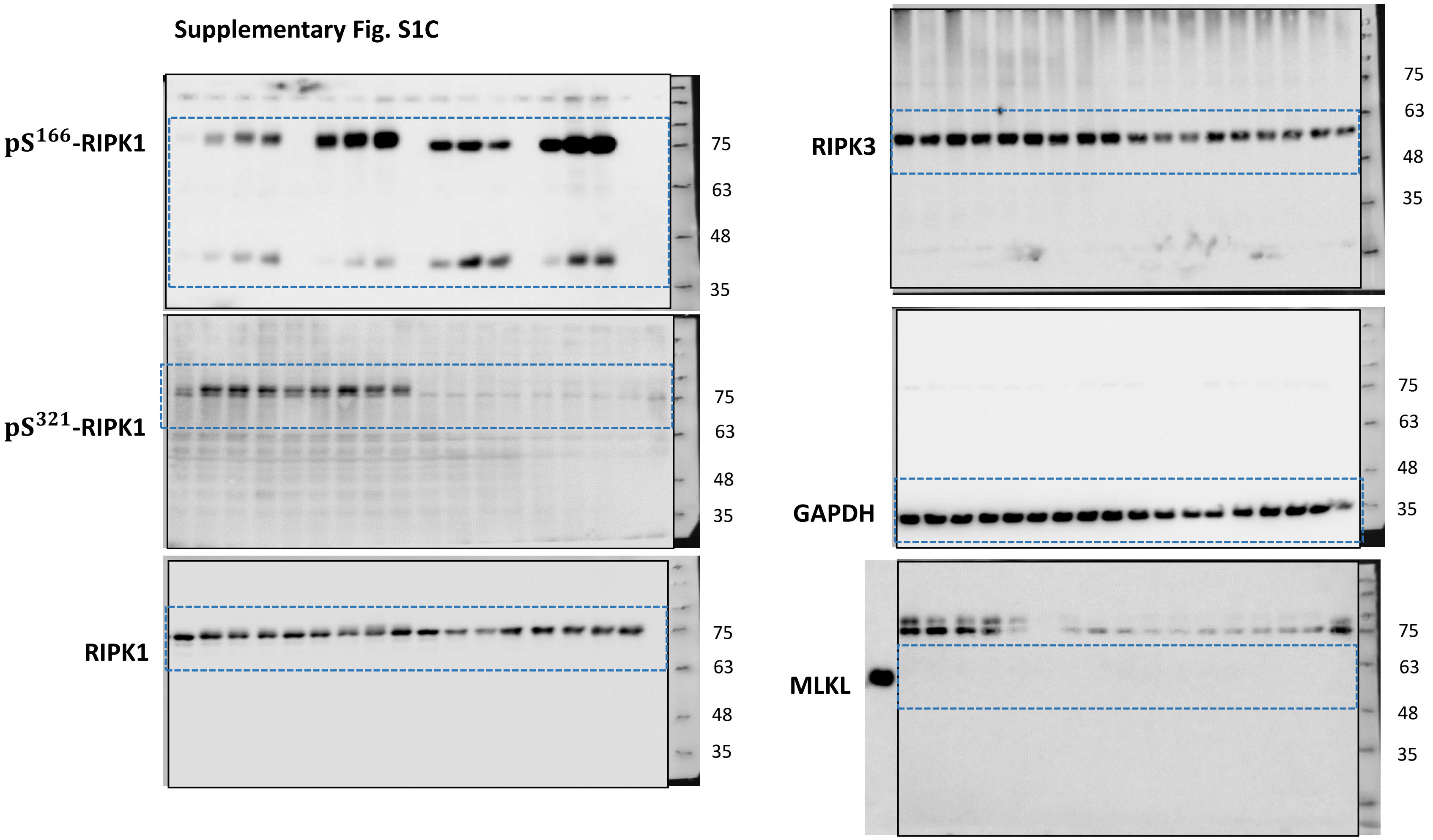

Supplementary Fig. S2

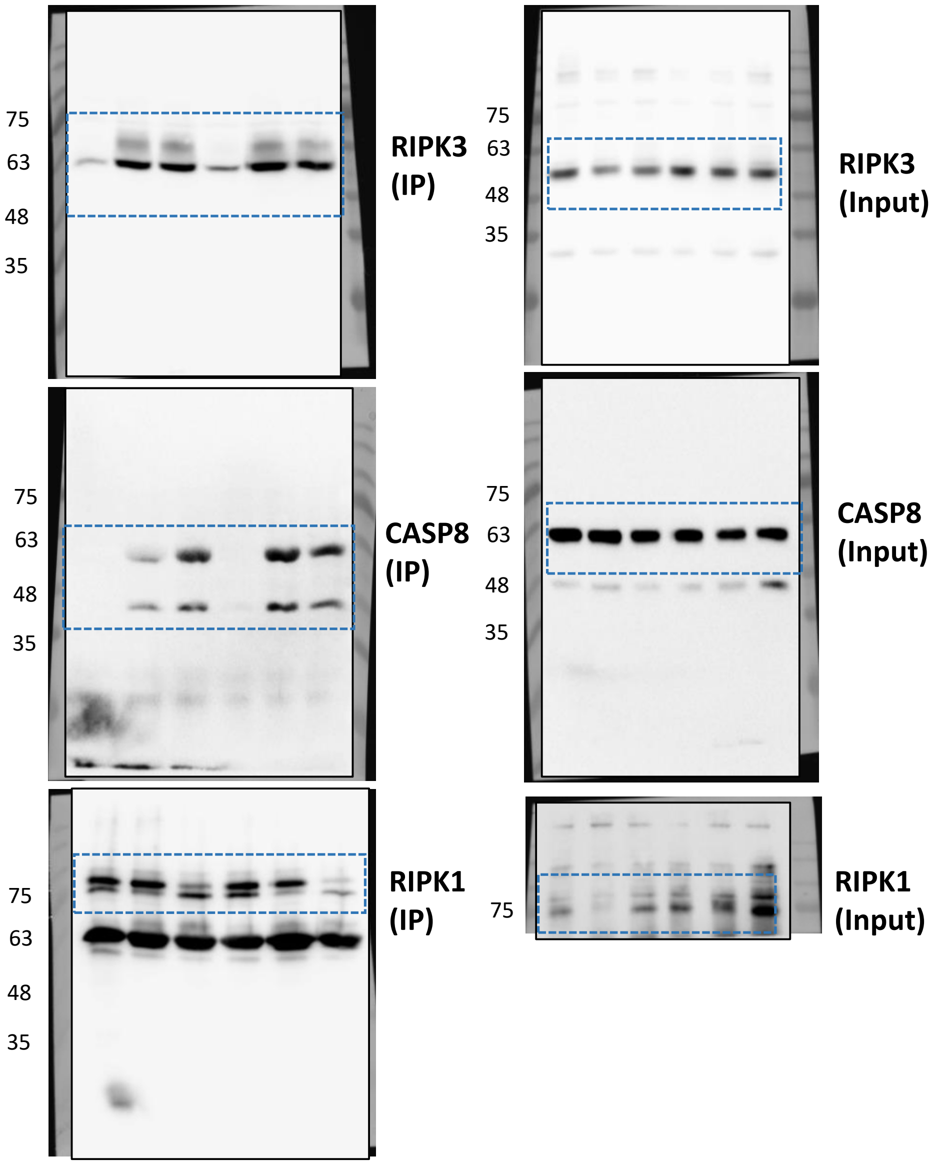

Supplementary Fig. S3

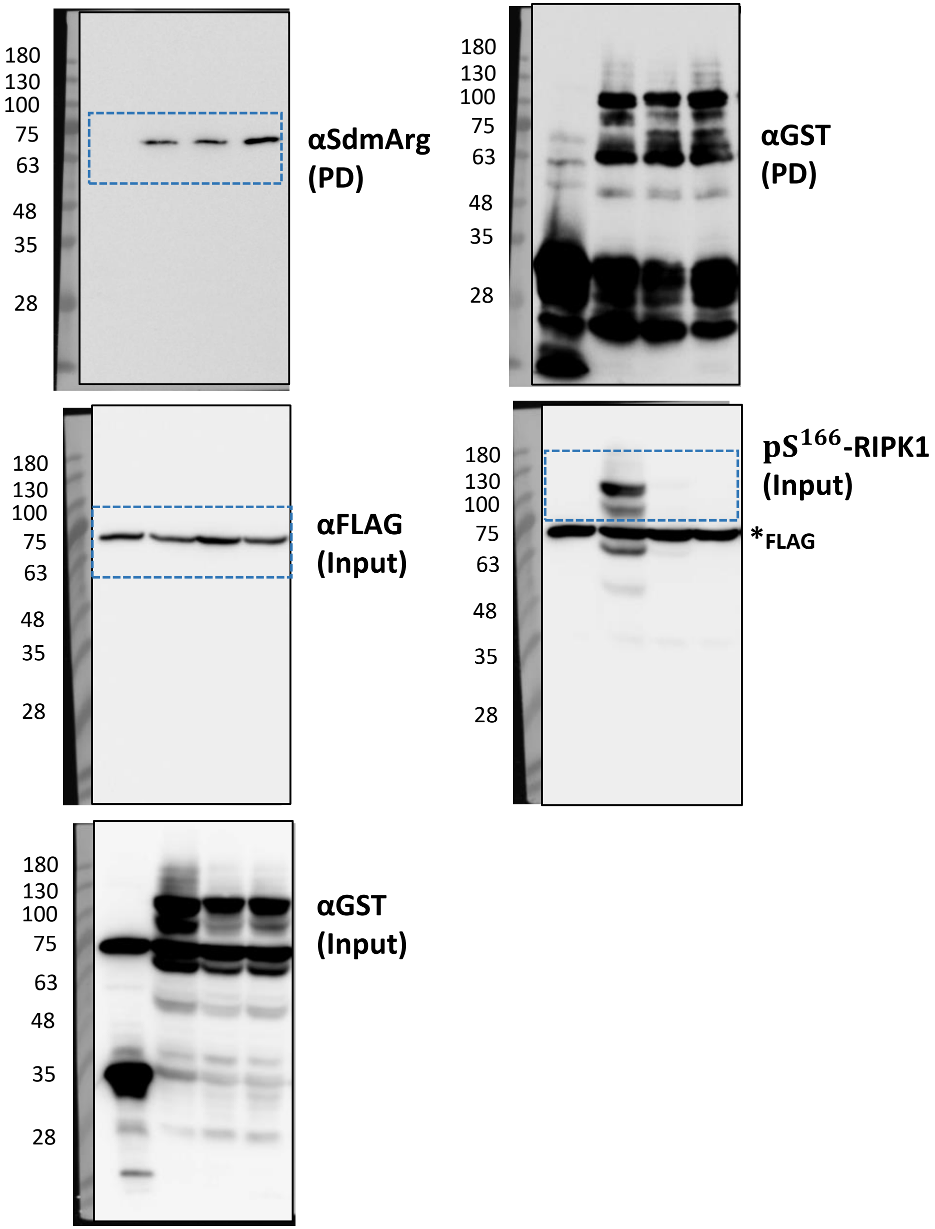

Supplementary Fig. S4

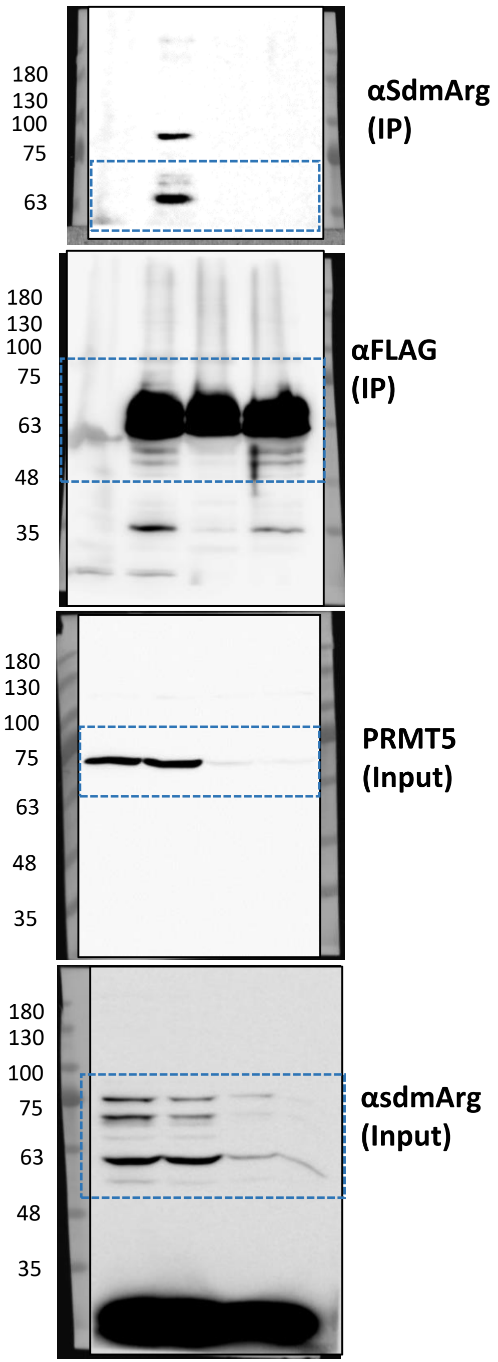

Supplementary Fig. S6

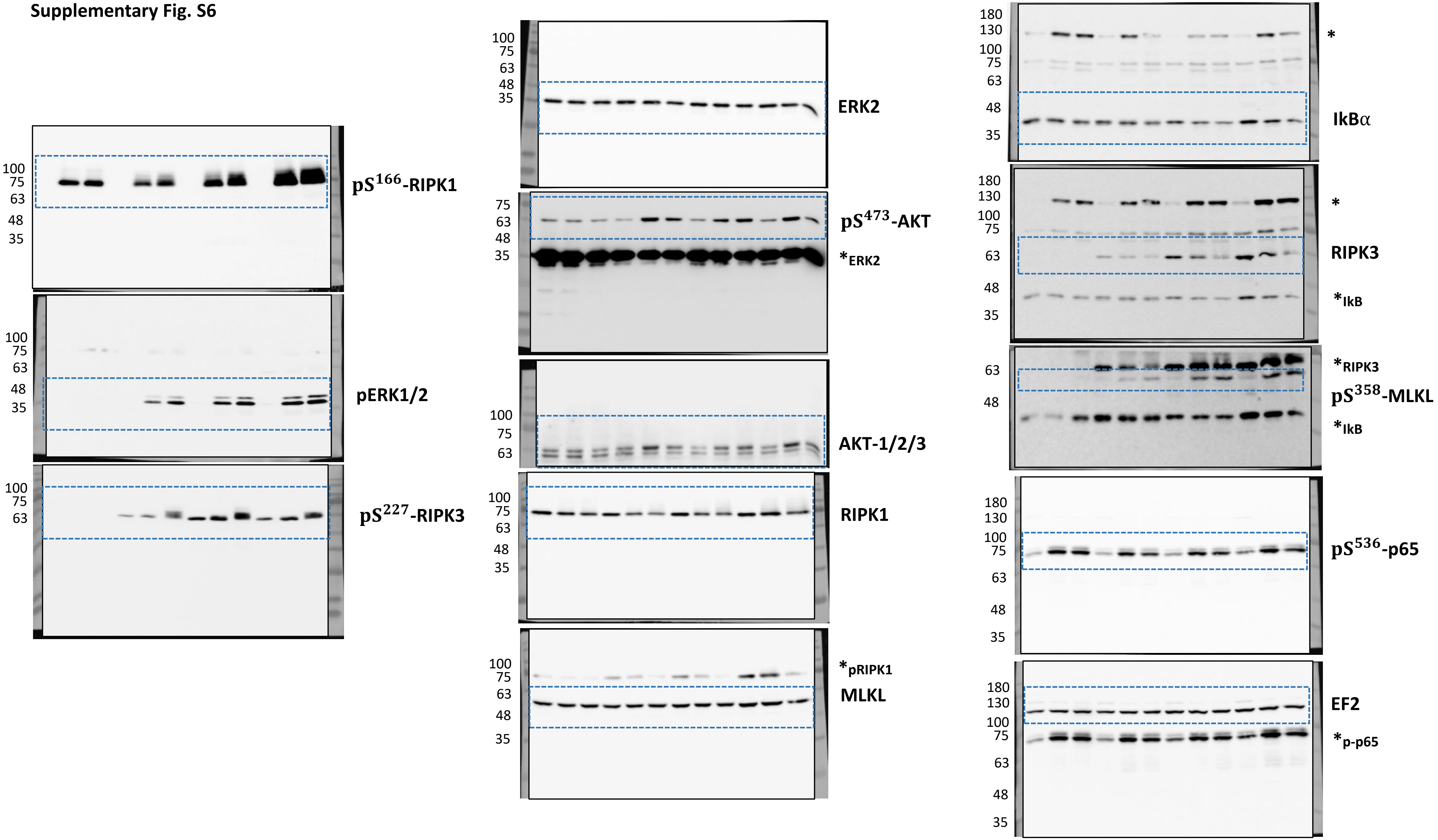

Supplementary Fig. S7

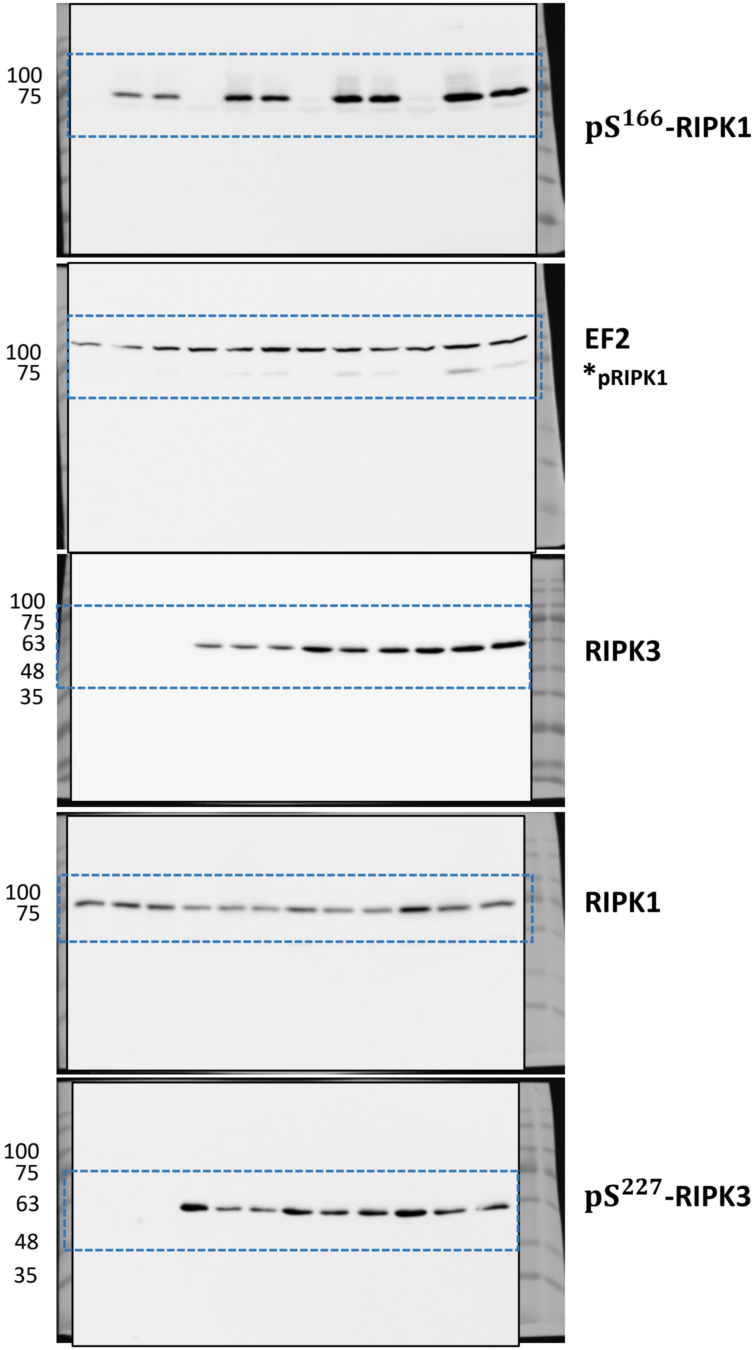

Supplementary Fig. S8

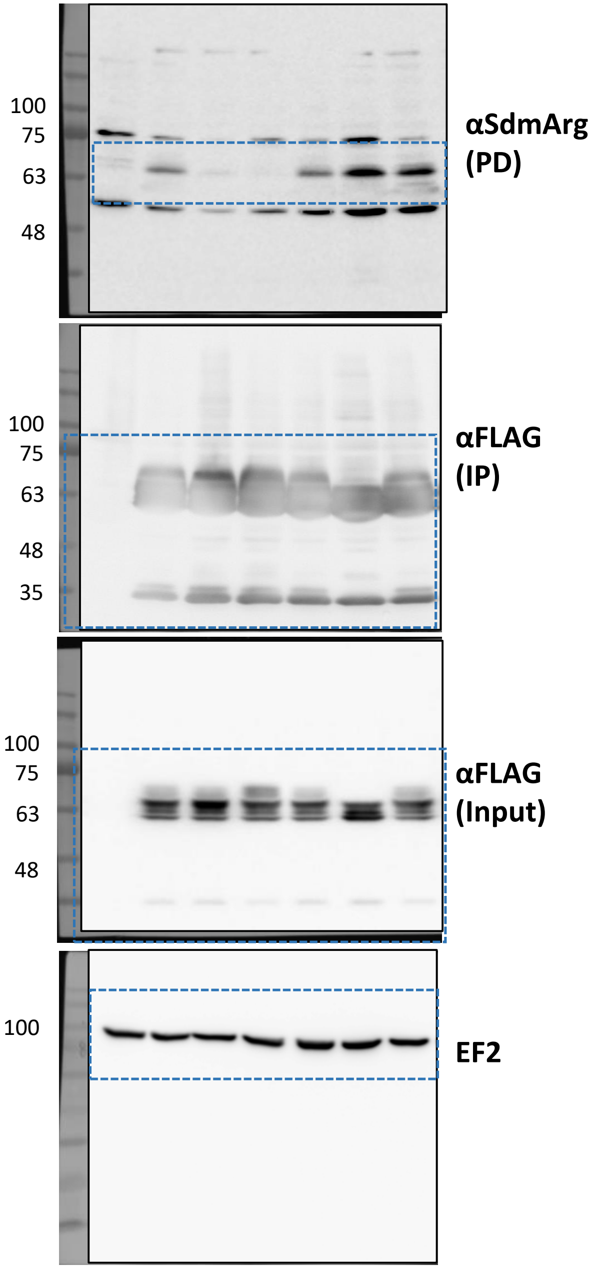

Supplement: Supplementary file 6 — Supplementary Information_Original Immunoblot data [file 41420_2023_1299_MOESM6_ESM.pdf]
